# Associations Between COVID-19 Symptoms and Psychological Distress

**DOI:** 10.3389/fpsyt.2021.721532

**Published:** 2021-08-17

**Authors:** Ju-Wan Kim, Hee-Ju Kang, Min Jhon, Seunghyong Ryu, Ju-Yeon Lee, Seung-Ji Kang, Sook-In Jung, Il-Seon Shin, Sung-Wan Kim, Robert Stewart, Jae-Min Kim

**Affiliations:** ^1^Department of Psychiatry, Chonnam National University Medical School, Gwangju, South Korea; ^2^Department of Infectious Diseases Medicine, Chonnam National University Medical School, Gwangju, South Korea; ^3^King's College London, Institute of Psychiatry, Psychology and Neuroscience, London, United Kingdom; ^4^South London and Maudsley NHS Foundation Trust, London, United Kingdom

**Keywords:** depression, anxiety, COVID-19 inpatients, insomnia, suicide idea

## Abstract

**Background:** Hospital isolation for COVID-19 may cause significant psychological stress. The association between COVID-19 symptoms and psychological symptoms has not been systematically studied. We investigated the effects of telephonic intervention on the relationship between psychological symptoms and COVID-19 symptoms at the time of hospitalization and 1 week later.

**Method:** We screened 461 patients with COVID-19 for psychiatric symptoms from February 29, 2020, to January 3, 2021. In total, 461 patients were evaluated 2 days after admission, and 322 (69.8%) were followed 1 week later. To assess anxiety and depressive symptoms, the Hospital Anxiety and Depression Scale (HADS) was administered to patients once per week. The Insomnia Severity Index (ISI) and item 9 of the Beck Depression Inventory (BDI-9) were used weekly to assess insomnia and suicidal ideation.

**Results:** Of 461 enrolled patients, we observed clinically meaningful psychological anxiety symptoms (in 75/16.3% of patients), depression (122/26.5%), insomnia (154/33.4%), and suicidal ideation (54/11.7%). Commonly reported COVID-19 symptoms are cough/sputum/sneezing (244, 52.9%), headache/dizziness (98, 21.3%), myalgia (113, 24.5%), and sore throat (89, 19.3%). Compared to baseline, significant improvements were found in anxiety, depression, and suicidal ideation at 1 week. No significant group differences in ISI score were observed.

**Conclusions:** COVID-19 symptoms at baseline had a significant and persistent negative impact on anxiety and depression at admission and at 1 week after hospitalization. Early intervention is essential to improve the outcomes of patients with mental illness.

## Introduction

The World Health Organization (WHO) declared the coronavirus disease 2019 (COVID-19) outbreak a pandemic on March 12, 2020 (1). Given the high contagion of COVID-19, many countries have adopted restrictive measures such as quarantines, lockdowns, and restrictions on movement and travel to contain the transmission. These can cause considerable psychological tension and difficulties in the general population (2). Psychological responses to infectious diseases include maladaptive behavior, emotional distress, and defensive reactions (3). In addition, previous studies have shown that inpatients with psychiatric problems have worse treatment outcomes and longer hospital stays (4, 5). Patients hospitalized for COVID-19 treatment face mental health issues of social isolation, loneliness, anxiety, depression, phobias, fear of disease progression, and insufficient resources at the time of admission (6). During hospitalization, patients continue to experience additional stresses related to COVID-19, creating new psychological problems that were not reported at the time of admission. In a meta-analysis of a recent studies of COVID-19 patients (including hospitalized and non-hospitalized patients), the prevalence of depression, anxiety, and sleep disorders was 45, 47, and 34%, respectively (7). Research on COVID-19 inpatients with psychiatric issues found that a considerable proportion of these patients reported depression (22.9–60.2%), anxiety (20.8–55.3%), and posttraumatic stress symptoms (96.2%) (8–12). However, these studies of psychological problems were single-timepoint investigations based on psychiatric assessments administered at difference timepoints, and they therefore provide only a snapshot of changing psychological symptoms.

The spectrum of COVID-19 symptom ranges from asymptomtic to critical; most infections are not severe (13, 14). Previous studies suggest that COVID-19 may affect the central nervous system through the associated inflammatory immune response and pharmacological drugs that are administered (15, 16). A chart review of hospitalized patients with COVID-19 (*n* = 214) found that central nervous system manifestations (e.g., dizziness, headache, or impaired cognition) occurred in 25% (17). However, the association between COVID-19 symptoms and psychological symptoms has not been systematically studied. Treatment is more difficult when physical diseases and mental problems are combined (18). Experts have underscored the need to provide psychological treatment for patients from the beginning of their isolation to minimize the impact of emotional distress. However, multidisciplinary care has not been available due to increased rates of infection and underestimates of the need for psychological intervention. Overall, acute COVID-19-related medical concerns and hospitalizations have increased the need for psychiatric approaches, but intervention has been limited.

Evidence-based interventions for psychological symptoms targeted at COVID-19 patients are scarce. Anmella *et al*. presented four clinical cases (delirium, psychotic symptom, anxiety, and depression) in COVID-19 inpatients and reported on the use of psychotropic medications to control psychiatric symptoms. However, it is difficult to generalize their results due to limitations of their study design. Recent research has focused on the use of online or telephonic psychological interventions to reduce the risk for infection-related emotional distress in COVID-19 (19, 20). However, the sample sizes have been relatively small, which limits the ability to detect real effects. Previously, we reported the results of telephone interventions with 33 COVID patients isolated in hospitals (20). Here, we used telephone interviews to evaluate psychological symptoms in COVID-19 patients at the time of hospitalization and 1 week thereafter (before discharge). The purpose of the telephone intervention was to provide education, empathy, encouragement, reassurance, and suggestions to quarantined COVID-19 patients, to reduce psychological symptoms and facilitate their adaptation to the ward environment. We also investigated the effects of telephonic interventions on the relationship between psychological symptoms and COVID-19 symptoms at the time of hospitalization and 1 week later.

## Methods

### Study Outline

**Figure 1:**
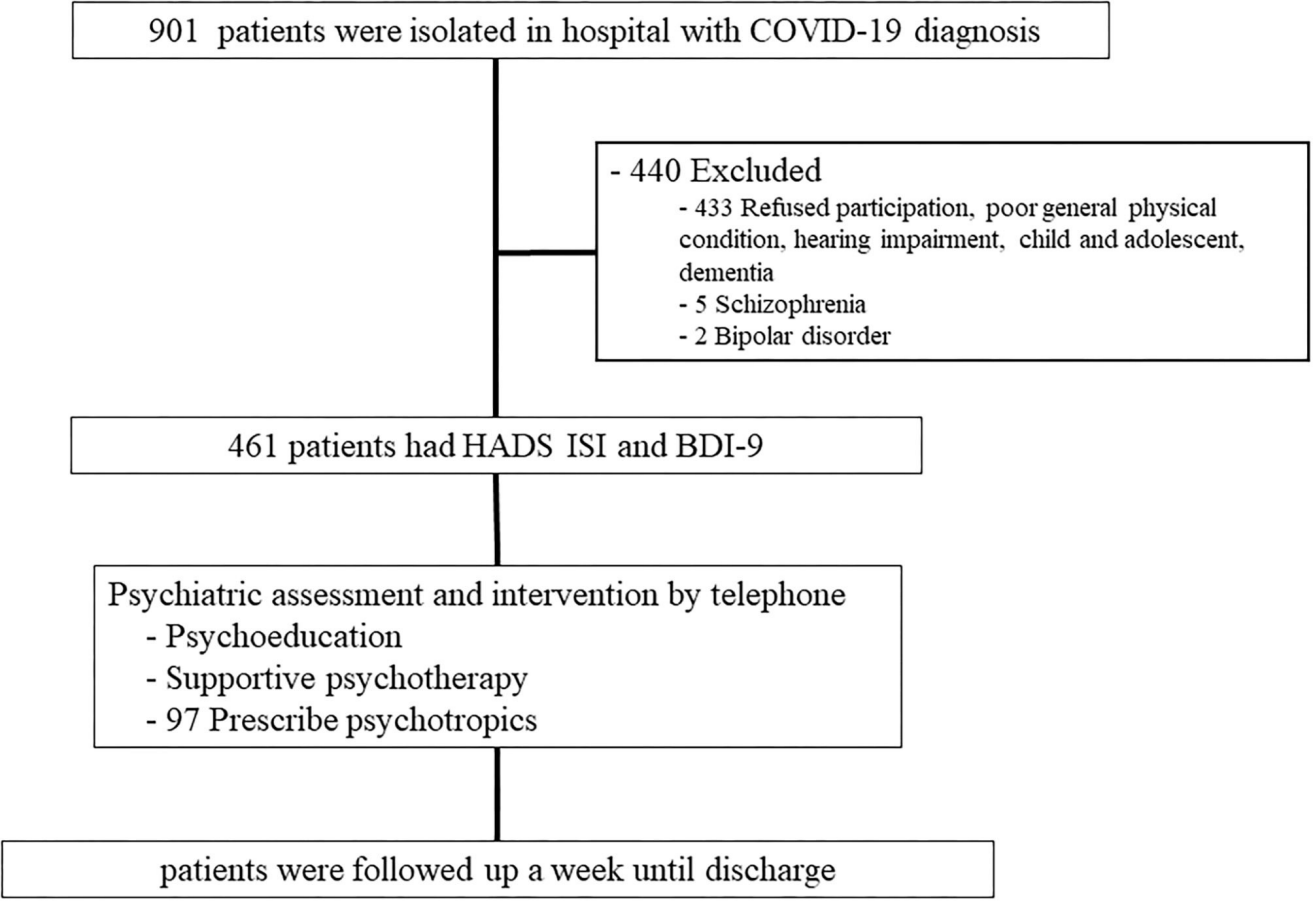
Flow chart of the study. HADS-A, Hospital Anxiety and Depression Scale- Anxiety; HADS-D, Hospital Anxiety and Depression Scale- Depression, ISI, Insomnia Severity Index; BDI, Beck Depression Inventory.

The Korean government classifies COVID-19 cases by severity to place priority on treating more severe cases at hospitals, while asymptomatic to mild cases receive medical treatment and monitoring at residential treatment centers. Patients who display COVID-19 symptoms receive conservative treatment and are discharged after clinical and testing criteria have been met. During the pandemic period, we developed a consultation network for patients with confirmed COVID-19 who were admitted to both types of hospital after consulting the infectious disease and psychiatry departments. Participants were consecutively recruited from hospitalized patients with COVID-19 at the Department of Infectious Diseases, Chonnam National University Bitgoeul Hospital (CNUBH), and Chonnam National University Hospital (CNUH), Gwangju, South Korea. The recruitment process is presented in Figure 1. Patients were screened at the time of hospitalization and 1 week thereafter (before discharge) using psychological scales related to anxiety, depression, suicidal ideation, and insomnia. The follow-up evaluation was performed 7.01 ± 0.8 days after the initial evaluation. After obtaining information on the scale scores, the study psychiatrists accessed the patients by telephone.

### Participants

Criteria for inclusion were as follows: a history of COVID-19 infection according to reverse-transcription polymerase chain reaction (RT-PCR) assay and hospitalization at CNUBH or CNUH for treatment of COVID-19 infection. Patients with severe communication problems (e.g., deafness, dementia, cognitive delay) were excluded. We screened 461 patients with COVID-19 for psychiatric symptoms from February 29, 2020, to January 3, 2021. This retrospective study strictly followed the code of ethics enforced by the Institutional Review Board of Chonnam National University Hospital (IRB No. CNUH-2021–020, IRB No. CNUH-2020–100). Written informed consent was waived by the ethics committee of the designated hospital for emerging infectious diseases.

### Measurement and Assessment of Psychiatric Symptoms

Psychiatric assessment was performed 2.04 ± 0.8 days after admission. The Hospital Anxiety and Depression Scale (HADS) was used to determine anxiety and depressive symptoms (21). The HADS was created to assess patients with a physical comorbidity using two seven-item subscales that evaluate depression and anxiety. A score of 8 was used as the cutoff point for each subscale to reflect a mild level of distress. The Insomnia Severity Index consist of seven questions assessing the impact of insomnia with a total score ranging from 0 to 28; a score of 8 was used as the cutoff point indicating a certain level of insomnia (22). Item 9 of the Beck Depression Inventory-II, which addresses suicidal thoughts or wishes, has a four-level response set. Suicidal ideation was considered present with a score ≥1 (23). The HADS, ISI and BDI-II were formerly translated and standardized in Korean (24–26). It was designated as creating a psychological difficulty due to COVID-19 when patients endorsed at least one question indicating depression, anxiety, insomnia, or suicidal ideation.

### Assessment of COVID-19 Symptoms and Clinical Characteristics

Clinical charts and nursing records were reviewed to investigate the absence/presence of COVID-19 symptoms. Clinical presentations of COVID-19 differ substantially and can include a range from asymptomatic infection through mild upper respiratory tract illness to severe viral pneumonia (27, 28). Although some clinical features (particularly smell or taste disorders) are more common with COVID-19 than with other viral respiratory infections (28), COVID-19 has no specific symptoms or signs. We investigated the presence or absence of nausea/anosmia/anorexia, headache/dizziness, fever/chills, chest discomfort/dyspnea, cough/sputum/sneezing, diarrhea, myalgia, and sore throat including symptoms from pre-hospital to psychological assessment. Individualized treatment strategies were used according to severity and the clinical setting. Severe COVID-19 was defined by the use of therapeutic drugs (steroid, antibiotics, and antiviral agents) excluding symptomatic drug use (29). C-reactive protein (CRP) was measured on the first day of hospitalization and within ±2 days. Sociodemographic data were collected on age, sex, alcohol, smoking, employment status, past medical history, and past psychiatric history.

### Psychological Intervention

Participants were approached about participating in the psychological intervention program, details of which are presented in Supplementary Table 1. Briefly, psychological interventions were performed at baseline and then once or twice per week until discharge. As a non-face-to-face intervention, telephone sessions with isolated patients focused more on psychoeducation to produce short-term effects. Beginning at admission, patients should be given sufficient information about the psychological effects of infectious diseases and isolation to reduce excessive fear. In addition, therapists can correct patients' inappropriate cognitive appraisals to reduce distress. To reduce the shortcomings of telephone access, psychiatrists implemented a telephone connection to the ward where the patients were hospitalized. By doing so, it was possible to collaborate for evaluation and treatment by sharing patients' information with the treatment team. For all patients admitted to the hospital, mental health guidelines that patients should know when in isolation were distributed. For patients who agreed to psychological intervention, a psychiatrist evaluated and counseled them twice a week for 5–30 min. Psychotropic medications were allowed if needed. Before and after the interventions, the patient's information was shared between the psychiatrists and medical treatment team to facilitate collaborative care.

### Follow-Up Evaluations for Psychiatric Symptoms

Each scale was administered once a week from the initial evaluation until discharge. After reviewing the scores on these scales, the psychiatrist provided treatment over the phone (routine follow-up, evaluation of psychological symptoms appearing after hospitalization, counseling regarding psychological problems, prescription of psychotropic medication, crisis intervention) according to the patient's symptoms.

### Statistical Analyses

Summary statistics are presented as means and standard deviations for continuous variables and as numbers and percentages for discrete variables. The demographic and clinical characteristics at baseline were compared by the presence or absence of COVID-19 symptoms using the *t*-test or the chi-square (χ2) test, as appropriate. The χ2 test was used to assess associations between sex or age and each scale score as a continuously distributed dependent variable. Suicidal ideation on follow-up was compared to baseline using a binomial exact test. Finally, the associations between COVID-19 symptoms and changes in psychological scales over 1 week were assessed using repeated-measures analysis of variance (ANOVA) in the same adjusted model. Statistical significance for all tests was set at *p* < 0.05. All statistical analyses were carried out using SPSS 23.0 software (IBM Corp., Armonk, NY, USA).

## Results

### Demographic and Clinical Characteristics

Of the initial 901 patients who were admitted for treatment of COVID-19, 461 subjects (192 male, mean age 50.42 ± 16.7, range 18–87 years) agreed to the psychological intervention described herein; their baseline sociodemographic and clinical characteristics are summarized in Table 1. At follow-up, 322 (69.8%) participants were reexamined. The mean duration of hospitalization was 12.5 days (±5.3). The remaining 139 (30.2%) patients were unable to complete the 1-week evaluation due to deterioration in COVID-19 symptoms, transfer to another hospital, or discharge from the hospital. Baseline HADS (D) scores were significantly lower in patients without than in patients with follow-up (5.2 ± 3.3 vs. 5.9 ± 4.0, *P* = 0.032). Fever was significantly less common in patients without than in patients with follow-up (38.5 vs. 24.5%). Otherwise, there were no substantial differences in any characteristics between participants who were and those who were not followed up (all *p* > 0.05).

**Table 1:** Baseline characteristics by common mental health problem as assessed using the HADS, BDI-9, and ISI at admission after COVID-19.

	**All participants (*N* = 461)**	**Psychological problemno(*N* = 226)**	**Psychological problem yes (*N* = 235)**	**Statistical coefficient**
**Socio-demographic characteristics**				
Age, mean (SD) years	50.42 (16.5)	50.64 (16.3)	50.20 (17.2)	*t* = +0.287 *P* = 0.775
Gender, N (%) female	269 (58.4)	113 (49.8)	156 (66.7)	χ^2^ = 13.520 ***P*** **<** **0.001**
Alcohol, N (%)	160 (34.7)	80 (35.2)	80 (34.2)	χ^2^ = 0.057 *P* = 0.812
Smoking, N (%)	56 (12.1)	28 (12.3)	28 (12.0)	χ^2^ = 0.015 *P* = 0.903
Currently unemployed, N (%)	175 (38.0)	74 (32.6)	101 (43.2)	χ^2^ = 5.459 ***P*** **=** **0.019**
**Psychiatric symptom characteristics**				
HADS (A), mean (SD) score	4.56 (3.5)	2.63 (2.1)	6.44 (3.6)	*t* = −13.969 *P* < 0.001
HADS (D), mean (SD) score	5.70 (3.8)	3.49 (2.0)	7.84 (3.9)	*t* = −15.196 *P* < 0.001
ISI, mean (SD) score	5.90 (5.2)	2.73 (2.3)	8.98 (5.3)	*t* = −16.379 *P* < 0.001
BDI-9, N (%)	54 (11.7)	0	54 (27.8)	χ^2^ = 54.956 *P* < 0.001
**COVID-19 symptom characteristics**, *N* (%)				
COVID-19 symptoms	167 (72.3)	158 (69.6)	186 (79.5%)	χ^2^ = 5.943 ***P*** **=** **0.015**
Nausea/ anosmia/ anorexia	40 (8.7)	16 (7.0)	24 (10.3)	χ^2^ = 1.496 *P* = 0.221
Headache/dizziness	98 (21.3)	38 (16.7)	60 (25.6)	χ^2^ = 5.454 ***P*** **=** **0.020**
Fever/chill	158 (34.3)	79 (34.8)	79 (33.8)	χ^2^ = 0.055 *P* = 0.814
Chest discomfort/dyspnea	38 (8.2)	15 (6.6)	23 (9.8)	χ^2^ = 1.581 *P* = 0.209
Cough/sputum/sneezing	244 (52.9)	110 (48.5)	134 (57.3)	χ^2^ = 3.587 *P* = 0.058
Diarrhea	44 (9.5)	20 (8.8)	24 (10.3)	χ^2^ = 0.279 *P* = 0.597
Myalgia	113 (24.5)	53 (23.3)	60 (25.6)	χ^2^ = 0.327 *P* = 0.567
Sore throat	89 (19.3)	35 (15.4)	54 (23.1)	χ^2^ = 4.338 ***P*** **=** **0.037**
Severe COVID-19	22 (10.0)	19 (8.4)	29 (12.4)	χ^2^ = 1.955 *P* = 0.162
CRP, mean (SD) mg/dL	1.29 (2.5)	1.25 (2.5)	1.32 (2.5)	*t* = −0.337 *P* = 0.737
**Past medical history**				
Psychiatric history	65 (14.1)	15 (6.7)	50 (21.7)	χ^2^ = 21.101 ***P*** **<** **0.001**
Physical history	211 (46.0)	102 (45.5)	109 (46.4)	χ^2^ = 0.033 *P* = 0.856
HTN	113 (24.6)	60 (26.8)	53 (22.6)	χ^2^ = 1.107 *P* = 0.293
DM	56 (12.2)	29 (12.9%)	27 (11.5%)	χ^2^ = 0.227 *P* = 0.634
Dyslipidemia	31 (6.8)	17 (7.6)	14 (6.0)	χ^2^ = 0.468 *P* = 0.494
Respiratory disease	32 (7.0)	11 (4.9)	21 (8.9)	χ^2^ = 2.865 *P* = 0.091
Rheumatic disease	12 (2.6)	7 (3.1)	5 (2.1)	χ^2^ = 0.448 *P* = 0.503
Endocrine disease	25 (5.6)	7 (3.1)	18 (7.7)	χ^2^ = 4.475 ***P*** **=** **0.031**
Heart disease	32 (7.0)	16 (7.1)	16 (6.8)	χ^2^ = 1.548 *P* = 0.214
Neurologic disease	25 (5.4)	12 (5.4)	13 (5.5)	χ^2^ = 0.007 *P* = 0.934

*Statistical coefficients were calculated using t-tests or χ^2^ tests as appropriate. Values in bold type show statistical significance. HADS-A, Hospital Anxiety and Depression Scale- Anxiety; HADS-D, Hospital Anxiety and Depression Scale- Depression, ISI, Insomnia Severity Index; BDI, Beck Depression Inventory*.

### Associations of COVID-19 Symptoms and Psychological Symptoms at Baseline

At the time of admission, the following clinically meaningful psychological symptoms were found: anxiety in 75 (16.3%) patients, depression in 122 (26.5%), insomnia in 154 (33.4%), and suicidal ideation in 54 (11.7%). Commonly reported COVID-19 symptoms were cough/sputum/sneezing (244, 52.9%), headache/dizziness (98, 21.3%), myalgia (113, 24.5%), and sore throat (89, 19.3%). The group with psychological symptoms (*N* = 235, 50.1%) were more likely to be female and jobless, to have COVID-19 symptoms of headache/dizziness and sore throat, to have a previous psychiatry history, and to have a diagnosis of previous endocrine disease. There were no significant differences between the groups in terms of alcohol use, smoking, CRP level, and severe COVID-19 status. Table 2 shows comparisons of anxiety, depression, insomnia, and suicidal ideation scale scores according to the absence/presence of COVID-19 symptoms. Baseline HADS (A) and HADS (D) scores were significantly higher in patients with COVID-19 symptoms, but the baseline ISI score did not differ significantly. There were no significant differences between the groups in BDI-9. Psychological symptoms at the time of admission according to age and sex are presented in Table 3. Women were more likely to have anxiety and depression symptoms than men (70.7 vs. 29.3%, 67.2 vs. 32.8%, respectively). There were no significant differences in psychological symptoms by age.

**Table 2:** Comparison of anxiety depression insomnia suicidal ideation among patients with/without COVID-19 symptoms.

	**All participants (*N* = 461)**	**No COVID-19 symptom (*N* = 117)**	**COVID-19 symptom (*N* = 344)**	**Statistical coefficient**	***P*-value**
HADS (A), mean (SD) score	4.56 (3.5)	3.94 (3.2)	4.77 (3.6)	*t* = −2.225	**0.027** ^*^
HADS (D), mean (SD) score	5.70 (3.8)	5.00 (3.7)	5.94 (3.8)	*t* = −2.324	**0.021** ^*^
ISI, mean (SD) score	5.90 (5.2)	5.28 (5.4)	6.11 (5.1)	*t* = −1.495	0.136
BDI-9, N (%)	54 (11.7)	9 (7.7)	45 (13.1)	χ^2^ = 2.452	0.117

* *P-value <0.05; ^†^P-value <0.01;^‡^P-value <0.001 by using t-tests, χ^2^ tests. Values in bold type show statistical significance. HADS-A, Hospital Anxiety and Depression Scale- Anxiety; HADS-D, Hospital Anxiety and Depression Scale- Depression, ISI, Insomnia Severity Index; BDI, Beck Depression Inventory*.

**Table 3:** Sex, age, and psychological symptoms in COVID-19 patients.

	**All participants (*N* = 461)**	**Anxiety (*N* = 75)**	**Depression (*N* = 122)**	**Insomnia (*N* = 154)**	**Suicide idea (*N* = 54)**
**Sex**					
Male	192 (41.6%)	22 (29.3%)	40 (32.8%)	53 (34.4%)	23 (42.6%)
Female	269 (58.4%)	53 (70.7%)	82 (67.2%)	101 (65.6%)	31 (57.4%)
Statistical coefficient		χ^2^ = 5.590 ***P*** **=** 0.018^*^	χ^2^ = 5.361 ***P*** **=** 0.021^*^	χ^2^ = 4.978 ***P*** **=** 0.026^*^	χ^2^ = 0.022 *P* = 0.881
**Age, years**					
≤ 20	9 (2%)	2 (2.7%)	2 (1.6%)	5 (3.2%)	2 (3.7%)
21–30	81 (17.6%)	13 (17.3%)	18 (14.8%)	29 (18.8%)	14 (25.9%)
31–40	40 (8.7%)	6 (8.0%)	15 (12.3%)	11 (7.1%)	5 (9.3%)
41–50	71 (15.4%)	8 (10.7%)	16 (13.1%)	18 (11.7%)	5 (9.3%)
51–60	113 (24.5%)	17 (22.7%)	30 (24.6%)	40 (26.0%)	11 (20.4%)
61–70	100 (21.7%)	21 (28.0%)	26 (21.3%)	38 (24.7%)	14 (25.9%)
71–80	38 (8.2%)	6 (8.0%)	14 (11.5%)	9 (5.8%)	3 (5.6%)
81–90	9 (2.0%)	2 (2.7%)	1 (0.8%)	4 (2.6%)	0
Statistical coefficient		χ^2^ = 0.441 *P* = 0.507	χ^2^ = 0.319 *P* = 0.572	χ^2^ = 0.034 *P* = 0.854	χ^2^ = 2.468 *P* = 0.116

* *P-value <0.05; ^†^P-value <0.01;^‡^P-value <0.001 by using t-tests, χ^2^ tests. Values in bold type show statistical significance*.

### Associations Between COVID-19 Symptoms and Psychological Symptoms at 1 Week

**Figure 2:**
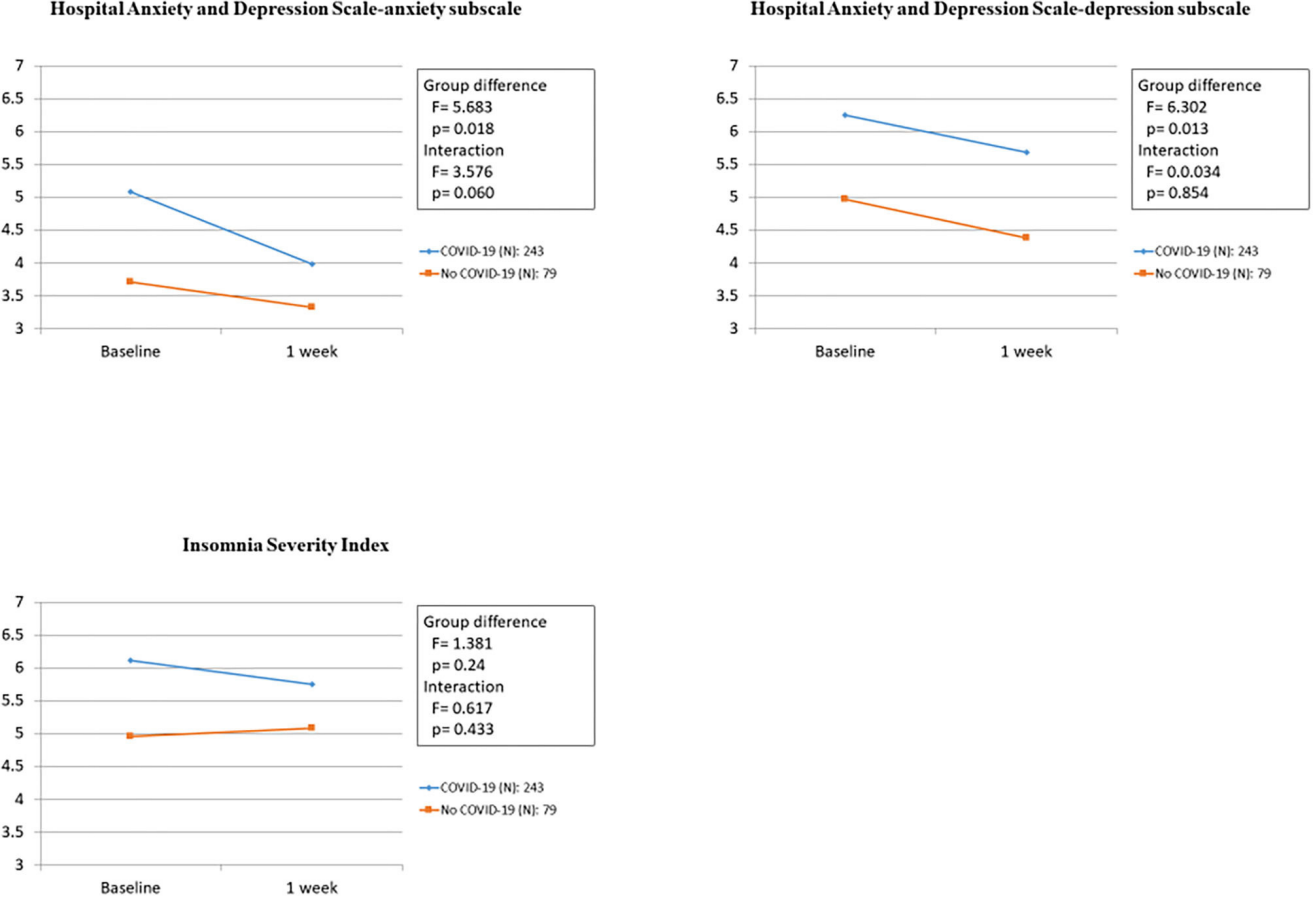
Associations of anxiety, depression, insomnia and COVID-19 symptoms at 1 week.

**Figure 3:**
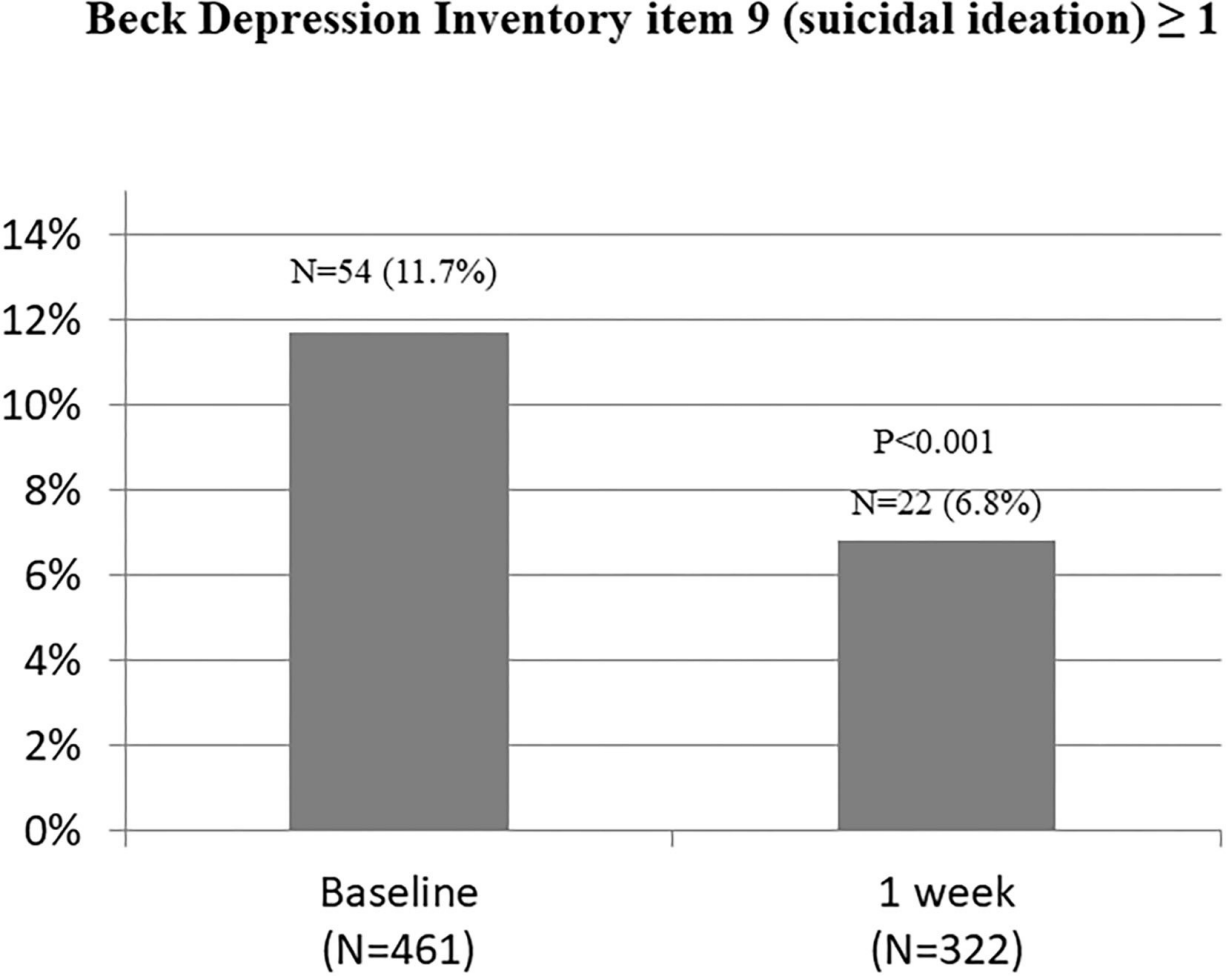
Changes in suicidal ideation from baseline to 1 week.

The associations between COVID-19 symptoms during admission and changes in the HADS (A), HADS (D), and ISI scores over 1 week in the 322 participants who completed the follow-up evaluation are shown in Table 4 and Figure 2. Compared to baseline, significant improvements were found in HADS (A) and HADS (D) scores at 1 week (*P* = 0.021 and *P* = 0.020, respectively). Changes in the HADS (A) and HADS (D) scores showed significant group differences after adjusting for sex, employment status, history of psychiatric problem, and history of endocrine disease. No significant group differences were observed for ISI scores. There was no significant group × time interaction for changes in the HADS (A), HADS (D), and ISI scores after adjusting for sex, employment status, previous history of psychiatric problems, and rheumatic disease. Figure 3 shows the changes in suicidal ideation between baseline and 1 week. Compared to baseline, significant improvements were found in suicidal ideation at 1 week. The impacts of psychiatric and physical comorbidities on the HADS (A), HADS (D), and ISI scores are shown in Supplementary Table 2. Changes in the HADS (A), HADS (D), and ISI scores showed significant time, group, and group × time interaction effects after adjusting for confounding variables. Participants with these comorbidities showed significantly greater decreases on various psychological measures.

**Table 4:** Associations of COVID-19 symptoms and psychological symptoms at 1 week.

**Measure**	**Group**	**Baseline**	**1 week**	**Baseline- 1 week**					
**Psychological measures**	**COVID symptoms**	**Mean (SD)**	**Mean (SD)**	**Time**		**Group**		**Interaction**	
				***F***	***P***	***F***	***P***	***F***	***P***
HADS (A),	Yes (*n* = 243)	5.08 (3.9)	3.98 (3.4)	5.357	**0.021**	5.683	**0.018**	3.576	0.060
	No (*n* = 79)	3.70 (2.9)	3.32 (3.0)						
HADS (D),	Yes (*n* = 243)	6.25 (4.0)	5.68 (4.0)	5.491	**0.020**	6.302	**0.013**	0.034	0.854
	No (*n* = 79)	4.96 (3.7)	4.37 (3.9)						
ISI	Yes (*n* = 243)	6.11 (5.2)	5.75 (5.3)	1.662	0.198	1.381	0.241	0.617	0.433
	No (*n* = 79)	4.95 (4.6)	5.08 (4.8)						

* *P-value <0.05; ^†^P-value <0.01;^‡^P-value <0.001 by using Repeated ANOVA. All data are adjusted for sex, unemployment, previous psychiatric history and endocrine disease. Values in bold type show statistical significance*.

## Discussion

The principal findings of this study indicate that psychological symptoms at baseline in COVID-19 patients are associated with female sex, lack of employment, previous psychiatric history and endocrine disease, headache/dizziness, sore throat, and the presence of COVID-19 symptoms. Moreover, the presence of such symptoms at baseline had a significant and persistent negative impact on anxiety and depression at admission and at 1 week after hospitalization.

The overall prevalence of depression, anxiety, sleep disturbances, and suicidal ideation among COVID-19 inpatients was 16.3, 26.5, 33.4, and 11.7%, respectively. The prevalence of depression, anxiety, insomnia, and suicide assessed at 1 week after hospitalization was 13.9, 27.6, 29.3, and 6.8%, respectively. Current evidence on the prevalence of mental illness in COVID-19 inpatients varies widely among studies. Previous studies have reported that a considerable proportion of patients exhibit depression (22.9–60.2%), anxiety (20.8–55.3%), and sleep disturbance (34%, CI: 19–50%) (7–12). The reasons for the difference in prevalence among studies are numerous. First, the screening tools used to assess psychological symptoms have a significant impact on the outcome prevalence values. For example, regarding depression assessment, PHQ-9 (with a cutoff of ≥5) showed similar prevalence rates at 52–53%. However, this prevalence estimate is substantially higher than the prevalence indicated by HADS-D at a cutoff of ≥8, which estimated the prevalence at 19–20% (7). Second, the prevalence depends on the current status of COVID-19 in the country where the study was conducted. The mortality rate and prevalence of COVID-19 affect people's psychology. In a recent psychological evaluation of COVID-19 inpatients in Bangladesh, the prevalence of depression and anxiety was reported as 87.3 and 55.7%, respectively (30). Third, it seems that differences in the time point of psychological evaluations in each study have an effect on the results. Patients were invited to participate in some studies at any time prior to discharge, whereas other papers failed to report the study participation process.

Psychological difficulties in the early phase of COVID-19 were associated with female sex, unemployed status, previous psychiatric history, endocrine disease, headache/dizziness, sore throat, and presence of COVID-19 symptoms. Unemployment, a risk factor that has not yet been investigated in COVID-19 patients, is likely a marker of low socioeconomic status, which is associated with a higher prevalence of depression (31). A psychiatric history is a risk factor for mental disorders such as depression, anxiety disorders, and schizophrenia. In this study, among patients hospitalized for COVID-19, those with a psychiatric history experienced psychological difficulties in the early stages of hospitalization (32, 33). We also found that females were at higher risk for psychological symptoms, which is consistent with previous findings that females were more susceptible to stress-related mental disorders, including depression in COVID-19 inpatients than males (11). The higher prevalence of depression in women is associated with hormonal changes and genetic loadings (34, 35). In general, dysregulation of the endocrine system, such as the thyroid and the hypothalamic–pituitary–adrenal axis, can lead to neuropsychiatric problems (32, 36). Considering these factors, patients with endocrine disorders may experience irregular and unpredictable mental states in early stages of COVID-19. Our results suggest that patients with endocrine disorders are more likely to have psychological problems when infected with COVID-19. However, because the number of patients with endocrine disorders in our study was small, follow-up studies related to this question should be performed. There is a significant association between the prevalence of physical symptoms and psychological distress among health-workers in a pandemic, which is probably bi-directional (37). However, the association between COVID-19 symptoms and psychological symptoms has not been systematically studied. Our research shows that the presence of COVID-19 symptoms is associated with the prevalence of psychological symptoms. In particular, among several COVID-19 symptoms, headache/dizziness and sore throat were associated with psychological symptoms. Furthermore, the presence of COVID-19 symptoms at baseline had a significant and persistent negative impact on anxiety and depression at admission and 1 week after hospitalization. It is also noteworthy that the emergence of symptoms at the time of hospitalization is greater when psychiatric and physical symptoms are present. These results suggest that the presence or absence of COVID-19 symptoms and degree of recovery also affect patient psychology. In the future, research on the mechanism by which these somatic symptoms affect patient psychology should be conducted.

Hospitalized patients who have been diagnosed with COVID-19 are vulnerable to mental health problems (8–12). Patients who are subjected to activity limitations, interpersonal disconnection, economic difficulties, and inadequate supplies while confined to a small hospital room may suffer from distress, including loneliness, disconnection, and anxiety. Furthermore, frustration and anger may increase as patients experience disappointment when their initial expectations are not met and they are forced to deal with long hospital stays and other people's negative responses to their COVID-19 infection. In summary, as the problems experienced by patients vary widely and change over time, it is crucial to evaluate changes in patients' conditions. Moreover, targeted multidisciplinary interventions are needed to support inpatients with COVID-19 by simultaneously addressing both the infectious disease and the psychological symptoms during hospitalization. We developed a consultation network for telephone intervention with COVID-19 patients. We found that telephone-based interventions significantly reduced psychological distress (anxiety, depression, suicidal ideation), except for insomnia, during the first week of hospitalization. The insomnia intervention was probably ineffective because the environment of isolation continues to have a negative impact. Activity restrictions, sudden changes in the sleep environment, and sharing space with other patients seem to be sufficient to induce insomnia in patients. Research and evidence-based guidelines targeting insomnia in inpatients with COVID-19 are needed.

One important consideration when treating patients by telephone involves allowing sufficient time for a thorough evaluation while not interfering with patients' other treatment modalities. To this end, mental health professionals and other members of the treatment team should share information to facilitate collaborative care. Such collaboration reduces unnecessary contact with the patient and enables mental health professionals to obtain information that is difficult to collect by telephone. In addition, the shortage of assessment time can be offset using appropriate psychometric measures. The Hospital Anxiety and Depression Scale, Insomnia Severity Index, and Beck Depression Inventory (item 9) are considered suitable brief evaluations of anxiety, depression, insomnia, and suicidal thoughts (18–21). When administered to patients isolated with COVID-19, these measures identify boredom, loneliness, anxiety, and insomnia due to hospitalization. However, therapists should further evaluate other psychological symptoms (e.g., guilt, anger, impulsivity, frustration, and so forth) that are not assessed by these formal measures. Patients with COVID-19 have reported feeling guilty and ruminating about whether they have infected their loved ones or others. Furthermore, human rights violations, which have frequently been cited in discussions of Korea's COVID-19 response, have been a major stressor for isolated patients. The exposure of personal information, including behavior prior to hospitalization, often angers and frustrates patients. Thus, therapists should evaluate psychological changes that cannot be evaluated by formal scales.

Limitations of this study were as follows. First, the findings are limited by the one-arm design and the single study site. This may limit the generalizability of the findings, although it facilitates consistency of evaluation and treatment. Findings were also drawn exclusively from a Korean population, and replication is needed in other ethnic groups. Second, our study, which did not include a control group, was unable to differentiate between the effectiveness of telephone based interventions and direct face-to-face interventions. However, we found that telephone-based interventions significantly reduced psychological discomfort in the first week of hospitalization. Third, a semi-structured interview method was not used in this study to evaluate psychological distress, although a semi-structured psychological intervention protocol was employed, as detailed in Supplementary Table 1. Due to the limited timeframe of this study, only one item of the BDI-II was used to evaluate suicide. In the future, it will be important to evaluate suicide in hospitalized patients with COVID-19 using a structured scale. Finally, research on mental health aspects of COVID-19 is still lacking, particularly in terms of the variables that can influence mental health outcomes.

## Conclusion

COVID-19 survivors may have a significantly higher rate of psychiatric problems (38). Early intervention is essential to improve outcomes in patients with mental illness. It is necessary to understand not only COVID-19 treatment but also its psychosocial effects. During the COVID-19 outbreak, patients isolated in hospitals manifest risk factors for, and symptoms of, psychosocial problems. Therefore, various intervention strategies are recommended to manage psychological problems in these patients.

## Data Availability Statement

The original contributions presented in the study are included in the article/Supplementary Material, further inquiries can be directed to the corresponding author/s.

## Ethics Statement

The studies involving human participants were reviewed and approved by IRB No. CNUH-2021–020, IRB No. CNUH-2020–100. Written informed consent for participation was not required for this study in accordance with the national legislation and the institutional requirements.

## Author Contributions

J-WK and J-MK had full access to all of the data in the study and take responsibility for the integrity of the data and the accuracy of the data analysis. S-WK and J-MK study concept and design. J-WK, S-JK, and S-IJ acquisition of data. J-WK, H-JK, RS, S-WK, and J-MK analysis and interpretation of data. J-WK and J-MK drafting of the manuscript. J-WK, RS, S-JK, S-IJ, S-WK, and J-MK critical revision of the manuscript for important intellectual content. J-WK, RS, S-WK, and J-MK statistical analysis. J-MK obtained funding. J-WK, MJ, SR, J-YL, and I-SS administrative, technical, or material support. S-WK and J-MK study supervision. All authors contributed to the article and approved the submitted version.

## Conflict of Interest

The authors declare that the research was conducted in the absence of any commercial or financial relationships that could be construed as a potential conflict of interest.

## Publisher's Note

All claims expressed in this article are solely those of the authors and do not necessarily represent those of their affiliated organizations, or those of the publisher, the editors and the reviewers. Any product that may be evaluated in this article, or claim that may be made by its manufacturer, is not guaranteed or endorsed by the publisher.
