# Supplementary material for: Associations Between COVID-19 Symptoms and Psychological Distress
Source: Front Psychiatry. 2021 Aug 17;12:721532. doi: 10.3389/fpsyt.2021.721532 (PMC8415963; doi:10.3389/fpsyt.2021.721532)
Supplement: Supplementary file 1 [file Data_Sheet_1.docx]

| **eTable 1. Telephone based intervention for COVID-19** | |
| --- | --- |
| **Objectives** | Reducing psychological symptoms and improving adaptation to the ward environment by providing education, empathy, encouragement, reassurance and suggestion to isolated patients with COVID-19 |
| **Baseline** | - Formation of therapeutic alliance: Providing a sense of care and support through empathy for COVID-19 infection and hospital isolation  - Providing mental health guideline for isolation/quarantine  - Psychological assessment  - Reassurance of excessive fear about COVID-19  - Prescribing psychotropic medication |
| **1week~ 5week** | - Formation of therapeutic alliance: Providing a sense of care and support through empathy for COVID-19 infection and hospital isolation  - Symptom follow-up and psychological assessment  - Consolation and encouragement for COVID-19 diagnostic test result  - Active listening and suggestion of ward adaptation difficulties/stress factor  - Cognitive reconstructions for irrational beliefs  - Prescribing psychotropic medication |
| **Before discharge** | - Reassurance and guidance for fear of re-infection  - Advice of psychological problems that may be experienced after discharge |

| **e Table 2. The impact of psychiatric and physical comorbidity of COVID-19 on HADS (A), HADS (D), and ISI scores** | | | | | | | | | |
| --- | --- | --- | --- | --- | --- | --- | --- | --- | --- |
| Measure | Group | Baseline | 1 week | Baseline- 1 week | | | | | |
| Psychological measures | COVID symptoms and psychological symptoms | Mean (SD) | Mean (SD) | Time | | Group | | Interaction | |
|  |  |  |  | F | P | F | P | F | P |
| HADS (A), | Yes (n= 128) | 6.55 (3.6) | 5.21 (3.5) | 9.135 | **0.003**^†^ | 79.598 | **<0.001^‡^** | 21.796 | **<0.001^‡^** |
|  | No (n= 194) | 3.19 (3.6) | 2.81 (2.8) |  |  |  |  |  |  |
| HADS (D), | Yes (n= 128) | 7.88 (4.2) | 7.42 (3.9) | 5.455 | **0.020**^*^ | 100.351 | **<0.001^‡^** | 5.432 | **0.020**^*^ |
|  | No (n= 194) | 4.20 (2.9) | 3.9 (3.4) |  |  |  |  |  |  |
| ISI | Yes (n= 128) | 8.91 (5.1) | 7.41 (5.3) | 4.759 | **0.030**^*^ | 63.561 | **<0.001^‡^** | 12.406 | **<0.001^‡^** |
|  | No (n= 194) | 3.84 (4.2) | 4.16 (4.6) |  |  |  |  |  |  |
| ^*^P-value<0.05; ^†^P-value<0.01; ^‡^P-value<0.001 by using Repeated ANOVA. All data are adjusted for sex, unemployment, previous psychiatric history and endocrine disease. Values in bold type show statistical significance. | | | | | | | | | |
